# Abundance decline in the avifauna of the European Union reveals cross‐continental similarities in biodiversity change

**DOI:** 10.1002/ece3.8282

**Published:** 2021-11-15

**Authors:** Fiona Burns, Mark A. Eaton, Ian J. Burfield, Alena Klvaňová, Eva Šilarová, Anna Staneva, Richard D. Gregory

**Affiliations:** ^1^ RSPB Centre for Conservation Science Cambridge UK; ^2^ RSPB Centre for Conservation Science Newcastle UK; ^3^ BirdLife International Cambridge UK; ^4^ Czech Society for Ornithology Prague Czech Republic; ^5^ RSPB Centre for Conservation Science Sandy UK; ^6^ Department of Genetics, Evolution and Environment Centre for Biodiversity & Environment Research University College London London UK

**Keywords:** assemblage, bird, conservation, Europe, population change

## Abstract

Although global assessments provide evidence of biodiversity decline, some have questioned the strength of the evidence, with local assemblage studies often showing a more balanced picture of biodiversity change. The multifaceted nature of biodiversity and imperfect monitoring datasets may partially explain these findings. Here, using an extensive dataset, we find significant biodiversity loss in the native avifauna of the European Union (EU). We estimate a decline of 17–19% in the overall breeding bird abundance since 1980: a loss of 560–620 million individual birds. Both total and proportional declines in bird numbers are high among species associated with agricultural land. The distribution of species’ population growth rates (ln) is centered close to zero, with numerical decline driven by substantial losses in abundant species. Our work supports previous assessments indicating substantial recent biodiversity loss and calls to reduce the threat of extinctions and restore species’ abundances, for the sake of nature and people.

## Graphical abstract

Using an extensive high‐quality dataset, we estimate a decline of 17%–19% in overall native breeding bird abundance in the European Union since 1980: a loss of 560–620 million individual birds. The distribution of species’ population growth rates (ln) is centred close to zero with numerical decline driven by substantial losses in abundant species. Our work supports previous assessments indicating recent biodiversity loss and calls to reduce the threat of extinctions and restore species’ abundances, for the sake of nature and people.

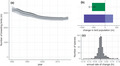

## 1 INTRODUCTION

Recent global assessments of environmental change highlight human‐driven loss of biodiversity and the degradation of ecosystem integrity (Díaz et al., 2019; Secretariat of the Convention on Biological Diversity, 2020). Further, they point to a failure to achieve existing biodiversity targets and call for transformative change across sectors of human society as an emerging Post‐2020 Global Biodiversity Framework takes shape (https://www.cbd.int/).

Yet at the same time, debate continues as to the nature and extent of biodiversity decline (e.g., Dornelas et al., 2019; Gonzalez et al., 2016; Leung et al., 2020; Vellend et al., 2017). That contrast in part reflects the inherent complexity of biodiversity and how temporal change can be measured. Different metrics capture different dimensions of biodiversity, are not necessarily correlated with each other, and differ in their suitability to measure biodiversity change, and some perform consistently better than others in that respect (Santini et al., 2017; Schipper et al., 2016). Recent attention has focused on the underlying pattern of change across species and on the extremes (Leung et al., 2020).

Although there is evidence for widespread declines from population surveys, assemblage surveys tend to suggest a more balanced picture of change (Dornelas et al., 2019; Vellend et al., 2013, 2017). To help understand those differences, Dornelas et al. (2019) suggested that the selection of study populations may be inadvertently biased toward declining species and questioned whether such “ideal” datasets do, or would ever, exist. Buckland and Johnston (2017) argue for monitoring programs to have (1) representative sampling locations; (2) sufficient sample sizes; (3) sufficient detections of target species; (4) a representative sample of species; and (5) a temporal sampling scheme designed to aid valid inference. In reality, few individual schemes meet all of these criteria, and this issue becomes compounded when data from multiple schemes are integrated to assess population change. Combining datasets can mean that a more representative sample of species is included but may introduce other potential biases, for example, due to differences in temporal coverage or sampling size.

Population studies of birds are some of the most well‐developed, reflecting the popularity of the taxa and the relative ease by which they are detected and identified (Gregory & van Strien, 2010). There is a rich tradition of bird surveys and atlas projects across the globe, often involving skilled amateur ornithologists with professionals in structured and well‐designed monitoring projects (Harris et al., 2020; Keller et al., 2020). Taking advantage of extensive high‐quality data, notably in the form of the North American Breeding Bird Survey, Rosenberg et al. (2019) modeled population change in 529 North American species (76% of breeding species) over 48 years. They integrated species’ population trajectories and population size estimates into a hierarchical Bayesian model to produce a time series of population sizes across species, estimating a decline of 29% in breeding bird abundance since 1970 and indicating a staggering net loss of 2.9 billion birds. Using the same data, Jörger‐Hickfang et al., 2020) estimated the proportional change per species and found a smaller average decline and suggested that biodiversity assessments should present a range, rather than a single measure of change.

Previous work using extensive data collected in Europe has also demonstrated substantial population declines in common and widespread birds, especially in those associated with agricultural systems (e.g., Donald et al., 2006; Gregory et al., 2005; Reif, 2013; Tucker & Heath, 1994) and in long‐distance migrants (Sanderson et al., 2006, 2016). Inger et al. (2015) combined annual trend estimates with estimates of population size for 144 widespread European birds (32% of native breeding species) between 1980 and 2009 to demonstrate a significant decline in the total bird abundance and biomass. Most of this loss was attributed to the more common species, whereas less abundant species showed an overall increase in both abundance and biomass.

Recent work has also emphasized the importance of species abundance as a key component and metric of biodiversity change. This is both from a perspective reflecting the intrinsic value of species and their persistence and, more broadly, the fundamental role that species populations play in the functioning of ecological systems and in the provision of ecosystem services, or goods, upon which humanity relies (Gaston, 2010; Mace et al., 2012). In that respect, common species are likely to contribute disproportionately more than rare ones as even relatively small proportional declines in the abundance of common species will result in large absolute losses of individuals and biomass, which may disrupt ecosystem structure, function, and services (Gaston & Fuller, 2008). For that reason, ambitions to recover depleted populations and restore the abundance of species are increasingly prominent in national and international environmental frameworks, for example, within the draft Post‐2020 Global Biodiversity Framework (United Nations Environment Programme, 2021).

In this study, we bring together two large avian datasets to explore abundance change in the European avifauna, taking the European Union (EU) as our geopolitical unit. Integrating these datasets allowed us to more than double the number of species included and to extend the temporal coverage by close to a decade compared with previous similar work. Our specific aim was to estimate change in the total population size of wild native breeding bird species in the EU and their average population growth rate, between 1980 and 2017, and to explore whether the previously observed patterns of change held true, given the extended taxonomic and temporal coverage.

We first test the hypothesis that the total avian abundance in the EU has not changed in the past 40 years, and whether the average population growth rate (log‐transformed) is negative. We then test the degree to which these patterns are associated with the abundance class of the bird species, their habitat affinities, and aspects of their ecology. We predict that (1) the overall avian abundance in the EU has fallen and the average population growth rate is negative; (2) that this decline is most marked in abundant bird species; (3) that declines have been most pronounced in birds associated with particular habitats (e.g., agricultural landscapes); and (4) that aspects of ecology, such as migration strategy, are associated with trends. Across species and categories, we examine the overall numerical change, positive and negative changes, percentage changes in populations, and annual rates of change because each captures a different aspect of biodiversity change.

As predicted, we find considerable numerical loss in the avifauna of the EU, although the rate of this decline has slowed and the underlying distribution of species’ changes is close to zero, and we find heterogeneity in patterns of change in different bird abundance categories, and in birds associated with different habitats consistent with past studies.

## 2 METHODS

### 2.1 Data collection and collation

Our analysis covers all breeding bird species native to countries in the EU where adequate data were available (86%; 378 species out of 445 native species that breed in the EU (European Commission, 2018).

#### 2.1.1 Annual time series

Annual population time series (annual index values and associated standard errors) for 169 common native European bird species (1980–2017) are derived from the Pan‐European Common Bird Monitoring Scheme (Brlík et al., 2020, 2021; www.pecbms.info). National time series, covering 26 of the 28 countries in the EU (as at the time, data were collated), are combined to produce a single EU‐level time series per species (EBCC/RSPB/BirdLife/CSO 2020). The Croatian monitoring program is being developed, and Malta currently lacks a national bird monitoring program. The United Kingdom is retained in the dataset as it was part of the EU throughout the period of analysis.

For a species’ EU‐level time series to be included in the dataset, the most recent year of the time series must represent at least 50% of the species’ current EU population. In some cases, however, the initial years of species’ time series are based on a small number of countries and/or represent a small proportion of the species’ EU population. Thus, when the time series are calculated, “missing” data are imputed from similar neighboring countries (Brlík et al., 2021). However, when very limited information is available, the resulting time series can have very wide confidence intervals, making estimates of population change across species very imprecise. Therefore, to reduce possible error, we chose to omit years for individual species where the species’ time series represented less than 5% of the species’ EU population (*N* = 37 species and 289 species’ years). Again to reduce uncertainty, we omitted two species entirely, *Anthus campestris* (tawny pipit) and *Galerida cristata* (crested lark), whose trends have been previously identified as being both imprecise and strongly negative (Gregory et al., 2019).

#### 2.1.2 Population trend and size estimates

Population trends (long‐term ~1980–2018 and short‐term ~2007–2018) and population estimates (~2013–2018) are available for bird species in the EU at a national level (Burns et al., 2021; Eionet, 2020). These data are collated every six years as part of mandatory reporting by EU Member States to the European Commission under Article 12 of the EU Birds Directive (2009/147/EC) (European Union, 2009). For the last two reporting rounds (2013 and 2019), BirdLife International was contracted by the European Commission to collate and validate these data, in close consultation with national experts. Following detailed guidance (DG Environment, 2017), each member state must report an estimate of the population size of each regularly occurring native breeding bird species and where possible associated trend estimates. Metadata are collected on the time period of each estimate; the method used, which varies from full or sampled surveys to expert opinion; and as a full set of references as possible. The data cover all 445 native breeding species in the EU. Both trends and population estimates, and the time periods they cover are most commonly represented by minimum and maximum estimates. On some occasions, a best single value is given in addition or instead of these. Most population estimates were expressed as numbers of pairs, with a small proportion as males, females, or individuals. In line with previous studies (Inger et al., 2015), we expressed all estimates as numbers of individuals, assuming one pair, one male, or one female equaled two individuals. Therefore, our results can be thought of as an approximation of the number of breeding individuals of native breeding bird species. The trend and population estimates may be based on (a) complete surveys or statistically robust estimates; (b) extrapolation from limited data; or (c) expert opinion with very limited data (DG Environment, 2017). Approximately 80% of the national population estimates and 70% of the national long‐term trends used were based on either option (a) or (b). Data quality varied by country and species, and although including lower quality data may introduce biases of its own, we decided to use all sources of population estimate and population trend data in this study in order for the taxonomic and geographic coverage to be as broad and representative of the full assemblage as possible.

Long‐term population trends were used in this study. The period over which the long‐term population trend was estimated varied between and within species but was ~1980–2018 in most cases; trend estimates were only included if they covered at least 16 years of this period. Trends were coded as increasing, decreasing, fluctuating around zero, or stable, with quantitative minimum and maximum trend estimates and/or best single value given for the first two categories and for some species/country combinations in the latter two categories. No overall change was assumed to have occurred where national species’ trends were coded as fluctuating, or as stable when no quantitative trend estimate was given.

Annual population time series across the EU were derived from national long‐term population trend estimates and population estimates. The proportion of a species’ EU population for which a trend estimate was available varied markedly. We included species for which a trend was available for at least a third of the EU population (*N* = 356; 80% of all 445 species). We used a lower cutoff for inclusion here compared with the 50% used for EU time series derived from national monitoring schemes (see Section 2.1.1) as the 50% threshold is for the most recent year and the average coverage across the time series is often lower. For each species in each country, we estimated the mean trend ($\bar{T}$; log scale), mean national population estimate (${\bar{E}}_{N}$: log scale), and mean year the population estimate was made ($\bar{y}$). Where a best single value was given, this was used. In the absence of a strict mathematical means to derive standard errors from maximum and minimum values, we roughly approximated standard errors around each mean estimate as a sixth of the difference between the maximum and minimum estimates (covering 99.7% of the distribution if it is assumed to be approximately normal). Where the population estimate maximum and minimum represented a 95% confidence interval, standard errors could be estimated more robustly as a quarter of the difference between the upper and lower estimate of the interval (where a 95% confidence interval is 1.96 standard errors above and below the mean). Where maximum and minimum values were absent, the standard error was set to zero.

For each species and each country, we used $\bar{T}$, ${\bar{E}}_{N}$, and $\bar{y}$, returning them to the measurement scale, where necessary, to estimate the population size in each year (*i*) 1980–2017 (Equation 1). The resulting country level time series were summed across countries in each year to obtain an EU‐level population time series for each species.

(1) $${\bar{E}}_{\mathrm{Ni}}={\bar{E}}_{N\bar{y}}\cdot \lambda^{\left(\iota ‐\bar{y}\right)};\;\text{where}\;\lambda ={\bar{T}}^{\left(1/\text{trend period}\right)}.$$

We used a bootstrap approach to estimate confidence intervals around the average time series. In each iteration (*N* = 100), the process described earlier was repeated using estimates of *T*, *E*, and *y* sampled from a normal distribution described by the average values and standard errors calculated before. The 2.5% and 97.5% quantiles of the bootstrap values for each year were taken as the lower and upper confidence limits.

#### 2.1.3 Linking annual time series to population estimates

Population size estimates were available for all species in each EU country, from Article 12 reporting. EU‐level species’ population estimates were calculated by taking the geometric mean of the summed minimum and maximum national species’ population estimates across countries, where only a best single value was available, and this was treated as both maximum and minimum. As before, we approximated the standard error around these estimates as a sixth of the span of the maximum and minimum values.

The period over which species’ national population estimates were made was approximately 2013–2018 (median start and end years, respectively), but there was variation within and between species. Nevertheless, only 2% of time periods started prior to 2000 and 10% prior to 2010. To obtain a single range estimate per species, we used the median of the country‐level start year estimates and the median of the country‐level end year estimates. We used these to estimate the midpoint and associated standard error, as described before.

#### 2.1.4 Covariates

Species were split into four quartiles of abundance, each containing an equal number of species, based upon their EU population estimate and labeled as rare, scarce, common, and abundant. We classified species according to their preferred breeding habitat following BirdLife International (2004), which is based on the habitat association matrix of Tucker and Evans (1997). The nine habitat associations were (1) marine; (2) coastal; (3) inland wetland; (4) tundra, mires and moorland; (5) boreal and temperate forests; (6) Mediterranean forest, shrubland and rocky habitats; (7) agricultural and grassland; and (8) montane grassland. Species that did not fit simply into these categories were labeled as (9) unclassified. A possible alternative description for this last group could be “generalists”; however, we felt that this implied a broad ecological niche, which might not always be the case where species do not fit well into a single one of the habitat classes used. We classified species to a migration strategy as (1) resident, (2) partial migrant, (3) short‐distance migrant, and (4) long‐distance migrant, following Sanderson et al. (2006). Species were split into four bird groups by family: landbirds, shorebirds, waterbirds, and waterfowl, following Rosenberg et al. (2019).

### 2.2 Estimating change in total avian population over time

A single EU‐level time series 1980–2017 was selected for each species, using those modeled on multiple national monitoring schemes, where available (*N* = 167; 2.1.1); otherwise, those derived from national trend and population estimates (*N* = 211; 2.1.2). The time series and the species’ EU population estimates were then analyzed using two different approaches described later, each with different statistical assumptions.

#### 2.2.1 Bayesian model

First, we used the Bayesian hierarchical model of Rosenberg et al. (2019; Smith, 2019) to estimate change in the abundance of EU birds. The model first creates smoothed species’ time series using a Bayesian GAM for time series modeled on multiple national monitoring schemes and then uses the species time series plus additional data (on breeding habitat and migration strategy) in a hierarchical Bayesian model that models both species‐ and group‐level trends in population size and shrinks uncertain species’ trends toward the group mean. This approach accounts for missing data at the start of species’ indices and incorporates uncertainty in both the annual estimates within the time series, and around the population estimate and the year of the population estimate. The value of initial missing years is set to that of the first year with data, and the variance associated with the missing values is increased by the square of the number of years since nonmissing data. This means that as the number of years between a missing estimate and the closest year with data increases, the estimate has less and less influence on the model output.

#### 2.2.2 Imputed model

The aforementioned approach shrinks uncertain species’ indices toward the group mean. This could be advantageous, but equally, it could introduce bias if species’ trends correlate with precision, which seems entirely plausible. To verify the earlier results, we used an imputed model like that of Inger et al. (2015), with a bootstrap approach to assess error. As before, the value of initial missing years was set to that of the first year with data. To estimate the average total abundance across species in each year (*A*), we expressed each species’ time series ($\bar{I}$) as a proportion of the value in the average population estimate year ($\bar{y}$), multiplied each time series value by the species’ average EU population estimate ($\bar{E}$ ) and summed across species (*s*) for each year (*i*) (Equation 2).

(2) $$A_{i}=\sum_{s=1}^{n}{\bar{I}}_{s,i}/{\bar{I}}_{s,\bar{y}}\cdot {\bar{E}}_{s,\bar{y}}.$$

We used a bootstrap approach (*N* = 1000) to create confidence intervals. In each iteration, we sampled *E*, *I* (both log scale), and y from a normal distribution and estimated the abundance across species in each year as before, after returning values to the measurement scale as necessary. The 2.5% and 97.5% quantiles of the bootstrap values for each year were taken as the lower and upper confidence limits. Our input data for the Imputed model were the same as for the Bayesian model, that is, including the species’ time series generated using the Bayesian GAM (2.2.1).

## 3 RESULTS

### 3.1 Overall population change in the EU avifauna

**FIGURE 1:**
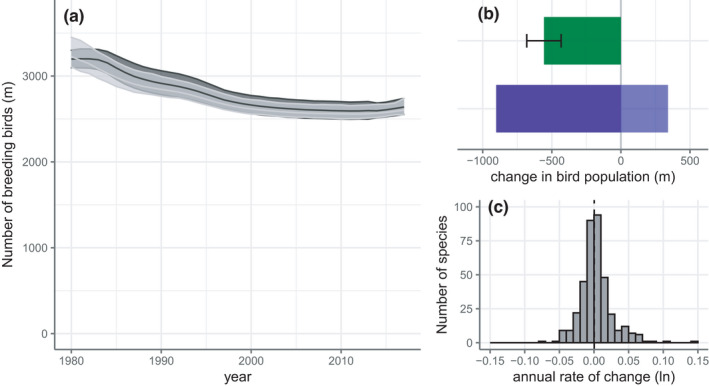
(a) Estimated total number of breeding individuals of native breeding bird species (millions) in the EU from 1980 to 2017, with shaded 95% credible intervals for the Bayesian model (dark gray) and 95% confidence intervals for the imputed model (light gray). (b) Overall net change with 95% credible intervals (green shading) and total increase among species with positive trends and total decrease among species with negative trends (blue shading). (c) Frequency distribution of species’ log‐transformed average annual rate of change. The dashed vertical line indicates the median value of the distribution

We estimate the total number of breeding individuals of native breeding bird species in the EU of the 378 species assessed to have declined by 557m individual birds between 1980 and 2017 (−17%; 95% credible interval −681 to −433) using the Bayesian model and by 623m birds (−19%; 95% confidence interval −803 to −468) using the imputed model (Figure 1a, Table 1). The estimated total abundance in 2017 was 2639 m (2551–2739) using the Bayesian model and 2603 m (2547–2739) using the imputed method.

**TABLE 1:** Total estimated number (millions), estimated change in number (millions, %, % per annum), of native breeding birds in the EU in 1980 and 2017, showing the 95% credible interval for the Bayesian model and the 95% confidence interval for the imputed model

Parameter	Bayesian model			Imputed model		
	Estimate	LCL	UCL	Estimate	LCL	UCL
1980	3197	3098	3300	3226	3106	3451
2017	2639	2551	2739	2603	2547	2739
Change	−557	−681	−433	−623	−803	−468
% Change	−17.42	−21.31	−13.54	−19.32	−24.90	−14.49
% per annum	−0.52	−0.63	−0.40	−0.58	−0.77	−0.42

Visual inspection indicates a difference in the rate of population decline in the late 20th compared with the early 21st century, with much of the decline in bird numbers occurring in the 1980s and 1990s. Piecewise regression on a log scale supports this, indicating a change in slope around the turn of the century (Bayesian: slope_1980:2001_: −1.00 (−1.04, −0.95), slope_2001:2017_: −0.029 (−0.10, 0.046), *R* ^2^ = 0.99; imputed: slope_1980:2000_: −1.01 (−1.07, −0.95), slope_2000:2017_: −0.029 (−0.11, 0.055), *R* ^2^ = 0.99), with a decline of approximately 1% per year in the first period and a rate of change not significantly different from zero thereafter.

Given the similarities in the output of the two modeling methods, hereafter, we report only the results of the Bayesian model (Table A1). There were no substantial differences between these findings and the equivalent results based on the imputed model (imputed model outputs are given in Table A2).

Given that some species have increased, the total decline across declining species was 903m and the total increase for increasing species was 341 m (Figure 1b). The distribution of log‐transformed species’ population growth rates was positively skewed (skew = 1.14) and leptokurtic (kurtosis = 7.57) (Figure 1c), with the central tendency close to zero (median: 0.00041).

### 3.2 Patterns of change by covariate

**FIGURE 2:**
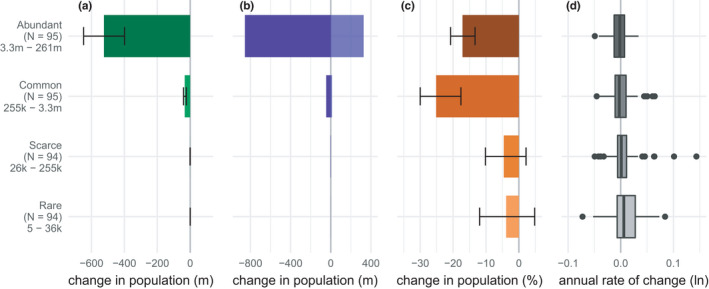
Patterns of change in native breeding bird species in the EU from 1980 to 2017 disaggregated by abundance category. (a) Net change in total abundance (millions of individuals) with 95% credible intervals. (b) Total increase in species with positive trends and total decrease in species with negative trends (millions of individuals). (c) Percent change in total abundance with 95% credible intervals. (d) Box plot of average annual rates of change across species on a log scale. The number of species followed by the range of species’ population sizes included in each category is given within brackets

As predicted, on average rare and scarce bird species in the EU avifauna have less negative population trajectories than more abundant species (Figure 2; Table A1). Overall, rare species showed a 4% decline in abundance as a group and scarce species a 5% decline (Figure 2c: rare % Δ_1980–2017_: −4 (−12, 5); scarce % Δ_1980–2017_: −5 (−10, 2)), whereas there was a 25% decline in the total abundance of common species (%Δ_1980–2017_: −25 (−30, −18)), and a 17% decline in abundant species (%Δ_1980–2017_: −17 (−21, −13)). The median log‐transformed population growth rate among rare species was 0.0059 and in scarce species 0.00098 (Figure 2d). In contrast, the average rate of change in common species was −0.0027 and in abundant species −0.0018 (Figure 2d).

The split between abundance categories is correlated with the method of time series derivation, with a higher proportion of rare and scarce species’ time series coming from national trends and population estimates than in common or abundant species. However, the trend toward abundant species declining proportionally more than rare species was apparent when all time series derived from national trends and population estimates, where considered separately (*N* = 356, Table A3).

**FIGURE 3:**
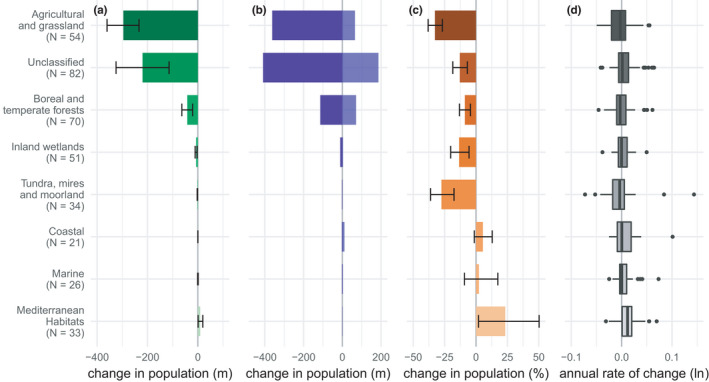
Patterns of change in native breeding bird species in the EU from 1980 to 2017 disaggregated by habitat category. (a) Net change in total abundance (millions of individuals) with 95% credible intervals. (b) Total increase in species with positive trends and total decrease in species with negative trends (millions of individuals). (c) Percent change in total abundance with 95% credible intervals. (d) Box plot of average annual rates of change across species on a log scale. The number of species in each category is given within brackets

The pattern of change in abundance varies with the species’ breeding habitat associations (Figure 3; Table A1). Only five species were associated with montane grassland, so this category was not plotted. We see the largest net decline among species associated with agricultural land and grasslands (Figure 3a; Δ_1980–2017_: −296 m (−361, −234)), followed by the “unclassified” species—those species not associated with any single habitat (Δ_1980–2017_: −220 m (−325, −114)). When summarized at a species level, the total decline across all declining species was similar between these two groups, but the total increase among species with positive trends was greater in the unclassified group (Figure 3b). The proportional change in the total abundance was greatest for species associated with agricultural land and grasslands (%Δ_1980–2017_: −33 (−38, −27)), as well as tundra, mires, and moorland (%Δ_1980–2017_: −28 (−36, −18). Species associated with coastal (Δ_1980–2017_: 0.2 m (0.0, 0.4), %Δ_1980–2017_: 5 (−1, 13)) and Mediterranean habitats (Δ_1980–2017_: 9 m (1, 20); %Δ_1980–2017_: 23 (2, 50)) saw small total increases in abundance. In the latter case, this represented a substantial proportional increase, given the low initial abundance of species associated with this habitat. The median log‐transformed population growth rate was negative for species associated with agricultural land and grasslands (Figure 3d, −0.0027), boreal and temperate forests (−0.0029), and tundra, mires, and moorland (−0.0038) but positive for those species associated with Mediterranean habitats (0.012).

Although resident and long‐distance migrant species show similar estimates of total loss over time, the lower total abundance of long‐distance migrants means they have declined proportionally more, although the credible intervals overlap (Table A1, long‐distance migrants: Δ_1980–2017_: −221 m (−270, −178), %Δ_1980–2017_: −33 (−39, −28); residents: Δ_1980–2017_: −214 m (−312, −121), %Δ_1980–2017_: −21, (−29, −13)). Among bird groups, Shorebirds show the largest proportional decline (Table A1, %Δ_1980–2017_: −38 (−44, −32)), whereas waterfowl show an increase (%Δ_1980–2017_: 23 (8, 40)).

### 3.3 Extreme patterns in species’ change

**FIGURE 4:**
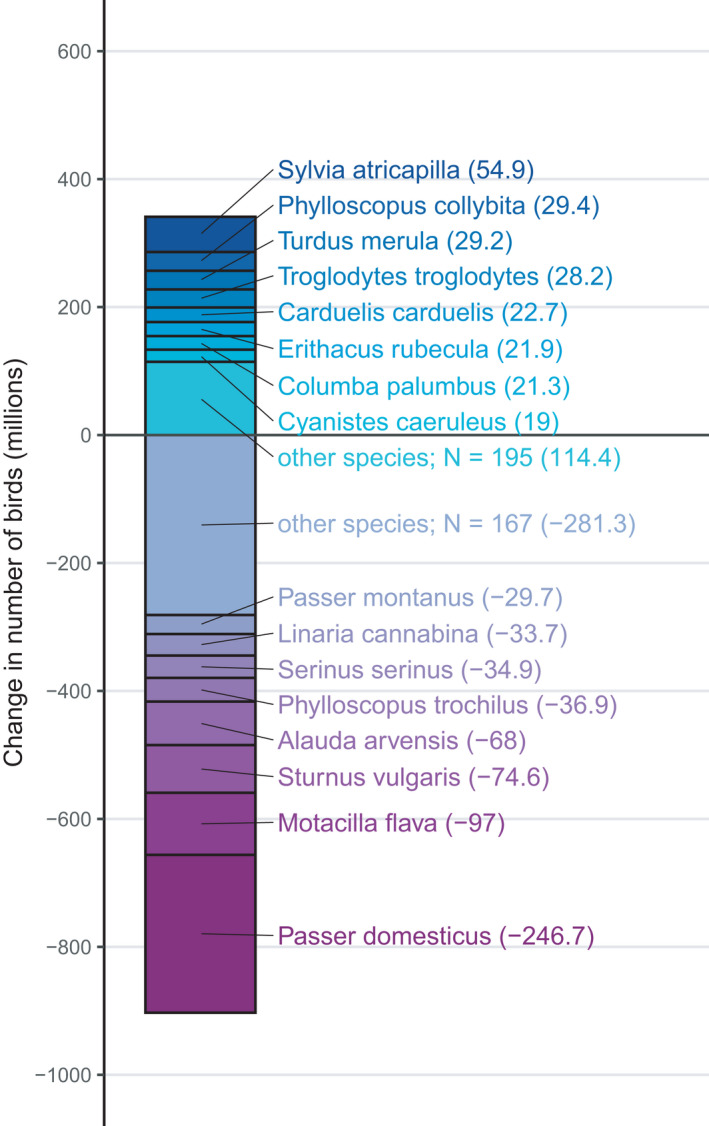
Change in bird species’ population size (millions of individuals) in the EU from 1980 to 2017. Species experiencing the eight largest numerical increases and the eight largest numerical decreases are named; other species are grouped as increasing or decreasing. *N* = number of species. Total change in population size is given within brackets

A small number of species were responsible for a large proportion of the change in numbers observed (Figure 4, species results file available in Burns et al., 2021) in both increasing and decreasing species. *Passer domesticus* (house sparrow) accounts for 27% (247 m) of the total decrease across all declining species. The eight species showing the largest declines account for 69% of the decline across all 175 declining species and the eight species showing the largest increases account for 66% of the increase across all 203 increasing species (Figure 4).

## 4 DISCUSSION

Using an extensive dataset and two analytical methods, we demonstrate significant biodiversity loss in the native avifauna of the EU. Specifically, we estimate a decline of 17%–19% in the overall bird abundance since 1980, which equates to a numerical loss of 560–620 m individual birds (Figure 1, Table 1). However, and at first counter‐intuitively, the average population growth rate of this assemblage is close to zero, and losses are driven by larger proportional declines in more abundant bird species (Figures 1 and 2). In addition, biodiversity loss is heterogeneous across habitats, with losses pronounced in birds associated with agricultural and grassland habitats, and to a lesser degree, in birds associated with Boreal and temperate forests (Figure 3). Long‐distance migrants and shorebirds also appear to have declined more strongly.

Our dataset, although imperfect, represents one of the best datasets of its kind, and we can be reasonably confident in a signal of overall biodiversity loss in the form of a large numerical loss in a bird assemblage at a near continental scale. However, the overall pattern of change conceals variation across species, abundance categories, and habitat. Debate has revolved around the balance between “winners” and “losers” in global biodiversity change (Dornelas et al., 2019; McKinney & Lockwood, 1999), but an equal balance of the two could result in biodiversity loss, or gain, if the magnitude of population changes on either side is not balanced. That is the case in our dataset, where the average population growth rate was close to zero, but the overall population change was negative. This argues, as others have, for a more nuanced view of biodiversity change (Dornelas & Daskalova, 2020; Dornelas et al., 2019; Leung et al., 2020). As in Leung et al. (2020), we also highlight the role of “extreme” population changes in driving the overall pattern of change, both increase and decline (Figure 4). In our case, a broad group of bird species have declined severely for a variety of reasons most closely associated with anthropogenic drivers of land use change (Donald et al., 2001, 2006), so although they are extreme in one sense, they represent a recognized characteristic of this assemblage and are measured with some precision.

Our results are strikingly similar to those of Rosenberg et al. (2019) in North America in showing substantial numerical losses, heterogeneity in trends among different groups of birds, and “extreme” changes in some species. Rosenberg et al. note that declines in North America parallel studies elsewhere and that the loss of native grassland birds is driven by habitat loss and pesticides, mirroring the loss of farmland birds in Europe (Bowler et al., 2019; Donald et al., 2006). Both studies identify the same groups of birds as of pressing conservation concern, namely, grassland/agricultural birds, shorebirds, and long‐distance migrants. Curiously, a small handful of super‐abundant species, including *Passer domesticus* (house sparrow) *and Sturnus vulgaris* (starling), drive the numerical decline, although these two species are only native in Europe. The North American dataset starts a decade prior to ours and shows a larger proportional decline in the total abundance. Large losses of farmland birds were reported from 1970 in Europe (Tucker & Heath, 1994); however, these changes varied spatially, with strong declines concentrated among countries in the west of the continent (Donald et al., 2001). Both studies also suggest a slowing in the rate of decline over the last decade. There is evidence that this change might be driven in part by conservation actions that have acted to protect species and create and restore habitats in North America and Europe. In the EU, the Birds Directive (2009/147/EC) and the Habitats Directive (92/43/EEC) provide legal protection to priority species and habitats (European Union, 1992, 2009) and have been shown to benefit target bird species (Donald et al., 2007; Sanderson et al., 2016) and enhance habitat protection (European Environment Agency, 2020). The impact of conservation may explain the trend toward rarer species having more positive annual growth rates (Figure 2). For example, seven of the species in the top decile of growth rates in our dataset are raptors (birds of prey). Many raptor populations have increased in recent decades following increased protection and reductions in pesticides and persecution, as well targeted species’ recovery projects (Deinet et al., 2013; Smart et al., 2010); a pattern mirrored in North America. The Natura 2000 network of protected sites created under the Birds and Habitats Directive has grown rapidly since the early 1990s, rising from 50,000 km^2^ in 1993 to 1,350,000 km^2^ in 2019; nevertheless, only 15% of habitats within the network are in good condition, and the network itself remains incomplete (European Environment Agency, 2020).

Taking our results more broadly, our study supports those reviews that indicate significant biodiversity loss over recent decades (Díaz et al., 2019; Secretariat of the Convention on Biological Diversity, 2020). The degree of loss in EU birds (−17%–19% in nearly 40 years) is similar to the Living Plant Index (LPI) for Europe and Central Asia (−24% average decline in vertebrate trends since 1970, (Almond et al., 2020)), to which many of the species’ time series used here contribute. Other studies describing biological loss in vertebrate populations globally have reported larger declines (Ceballos et al., 2015, 2017). It is argued that historical losses due to land conversion and degradation have left many temperate populations highly depleted, so modern‐day trajectories of species in some temperate and tropical comparisons are likely to be different (Newbold et al., 2016). Thus, although we would argue that our dataset is one of the best of its kind available globally, and is a good representation of the EU avifauna, we are not able to generalize these results to other taxa in this region, or to other bird populations and taxa in different parts of the world. Improvements in global monitoring efforts are needed to support similar assessments for different taxonomic groups or regions (Moussy et al., 2021).

The numerical loss of common and abundant species is a concern as it implies damage to the ecosystem structure and function and potentially to the delivery of ecosystem services. Common species may have a lower, higher, or equivalent influence per capita on ecosystem services, but their numerical dominance means that changes in their populations may have large impacts on service provision (Gaston, 2011). For instance, the abundance of common plant species has been seen to strongly influence primary productivity (Smith & Knapp, 2003). Most functional relationships between ecosystem service provision and bird abundance are positive (Gaston et al., 2018). For example, the number of a pest insect species consumed increases with increasing bird numbers (Crawford & Jennings, 1989), as does tree seed recruitment in areas of forest regeneration with increased abundance of frugivorous birds (Martínez & García, 2017), and people living in areas of higher bird abundance have lower levels of stress, anxiety, and depression, although effect sizes were low (Cox et al., 2017).

## 5 CONCLUSIONS

Here, we demonstrate substantial biodiversity loss using a comprehensive dataset at an assemblage level in the EU. Patterns of change also vary with the abundance class of the species, the habitat they frequent, and other aspects of their ecology, in close parallel to recently described patterns of change in North America. The consistency of patterns of loss across continents makes clear the urgent conservation needs of birds associated with agricultural land/grassland and long‐distance migrants. Large declines in the total avian abundance in the EU hide variation in terms of net and gross change in populations, proportional change, and average per annum rates of change. We argue that careful assessment of biodiversity change is needed to understand how to respond in policy terms to the emergent patterns.

Our results support the draft Post‐2020 Global Biodiversity Framework of the Convention on Biological Diversity (United Nations Environment Programme, 2021), which calls for increasing conservation efforts to be targeted toward preventing global and national extinctions, and the need to stabilize and restore globally and nationally depleted populations. For the latter to be successful, we need large‐scale conservation actions to be implemented widely and effectively across a range of biomes. There is great potential for mechanisms within the EU Biodiversity Strategy 2030 and specifically through the proposed EU ‘restoration law’ to define legally binding targets to restore habitats and species and drive this endeavor. This would require transformative actions cross sectors to tackle the nature and climate crises in tandem: protected area networks, species protection, nature‐friendly farming, forestry, and fisheries are all key parts of wider society solutions. The datasets used here will play an important role in monitoring the impact of these and related conservation actions.

## CONFLICT OF INTEREST

None declared.

## AUTHOR CONTRIBUTIONS

**Fiona Burns:** Conceptualization (equal); Formal analysis (lead); Methodology (lead); Writing‐original draft (equal); Writing‐review & editing (equal). **Mark A. Eaton:** Conceptualization (equal); Methodology (supporting); Writing‐review & editing (equal). **Ian J. Burfield:** Data curation (equal); Methodology (supporting); Writing‐review & editing (equal). **Alena Klvaňová:** Data curation (equal); Methodology (supporting); Writing‐review & editing (equal). **Eva Šilarová:** Data curation (equal); Methodology (supporting); Writing‐review & editing (equal). **Anna Staneva:** Data curation (equal); Methodology (supporting); Writing‐review & editing (equal). **Richard D. Gregory:** Conceptualization (equal); Methodology (supporting); Writing‐original draft (equal); Writing‐review & editing (equal).

## Data Availability

The two input datasets and the species’ level results have been archived on Zenodo (Burns et al., 2021).

## APPENDIX 1

**TABLE A1:** Total change (millions of individuals) and proportional change in breeding bird numbers in the EU, estimated using the Bayesian hierarchical model (see Section 2.2.1) and showing the median estimate and the lower and upper bounds of the 95% credible interval in each case, disaggregated by abundance category, bird group, migration strategy and breeding habitat

Grouping	Category	*N*	Total change (m)			Proportional change		
			Median	LCI	UCI	Median	LCI	UCI
All		378	−557.01	−681.47	−432.76	−0.174	−0.213	−0.135
Abundance category	Abundant	95	−524.11	−647.53	−398.89	−0.171	−0.207	−0.133
	Common	95	−33.02	−40.30	−23.00	−0.252	−0.300	−0.176
	Scarce	94	−0.44	−1.02	0.21	−0.046	−0.102	0.022
	Rare	94	−0.03	−0.12	0.04	−0.039	−0.119	0.048
Bird group	Landbirds	255	−548.65	−673.22	−424.34	−0.174	−0.209	−0.137
	Shorebirds	31	−6.13	−7.29	−4.95	−0.384	−0.441	−0.321
	Waterbirds	64	−3.69	−5.35	−1.95	−0.165	−0.227	−0.089
	Waterfowl	28	1.60	0.59	2.83	0.226	0.080	0.402
Migration strategy	Long‐distance migrant	115	−221.17	−269.57	−178.29	−0.332	−0.385	−0.279
	Partial migrant within Europe	127	−116.57	−182.24	−49.83	−0.078	−0.120	−0.034
	Resident	81	−213.65	−312.15	−120.93	−0.213	−0.293	−0.125
	Short‐distance migrant	55	−4.15	−8.30	0.28	−0.118	−0.225	0.008
Breeding habitat	Agricultural and grassland	54	−295.90	−360.57	−233.62	−0.327	−0.381	−0.268
	Boreal and temperate forests	70	−42.15	−63.60	−20.74	−0.089	−0.132	−0.044
	Coastal	21	0.17	−0.04	0.38	0.055	−0.014	0.129
	Unclassified	82	−219.61	−324.91	−114.25	−0.129	−0.185	−0.069
	Inland wetlands	51	−7.05	−10.82	−2.86	−0.134	−0.201	−0.055
	Marine	26	0.19	−0.84	1.46	0.024	−0.092	0.174
	Mediterranean habitats	33	9.37	0.88	19.66	0.233	0.020	0.503
	Montane grassland	5	0.46	0.27	0.71	0.646	0.360	1.001
	Tundra, mires, and moorland	34	−1.90	−2.61	−1.18	−0.275	−0.362	−0.175
	Unknown	2	0.001	−0.0005	0.003	0.318	−0.128	0.961

**TABLE A2:** Total change (millions of individuals) and proportional change in breeding bird numbers in the EU, estimated using the imputed model (see Section 2.2.2) and showing the deterministic estimate and the lower and upper bounds of the 95% confidence interval in each case, disaggregated by abundance category, bird group, migration strategy, and breeding habitat

Grouping	Category	*N*	Total change (m)			Proportional change		
			Estimate	LCI	UCI	Estimate	LCI	UCI
All		378	−623.18	−803.24	−467.52	−19.32	−24.90	−14.49
Abundance category	Abundant	95	−583.95	−769.75	−431.16	−18.91	−24.92	−13.96
	Common	95	−38.79	−70.98	−0.96	−30.23	−55.32	−0.75
	Scarce	94	−0.45	−0.96	0.14	−5.49	−11.66	1.74
	Rare	94	0.01	−0.06	0.05	1.00	−8.62	6.92
Bird group	Landbirds	255	−613.58	−804.77	−450.84	−19.30	−25.31	−14.18
	Shorebirds	31	−6.39	−7.83	−5.20	−39.81	−48.78	−32.39
	Waterbirds	64	−4.87	−7.22	−2.92	−20.88	−30.95	−12.49
	Waterfowl	28	1.66	0.75	2.67	23.51	10.60	37.79
Migration strategy	Long‐distance migrant	115	−255.75	−374.05	−182.55	−36.99	−54.11	−26.41
	Partial migrant within Europe	127	−133.46	−205.99	−65.63	−8.97	−13.85	−4.41
	Resident	81	−228.74	−357.64	−124.08	−22.61	−35.36	−12.27
	Short‐distance migrant	55	−5.23	−10.85	−0.36	−14.53	−30.14	−1.01
Breeding habitat	Agricultural and grassland	54	−331.36	−443.21	−249.87	−35.65	−47.69	−26.89
	Boreal and temperate forests	70	−48.07	−70.19	−26.27	−10.09	−14.74	−5.52
	Coastal	21	0.13	−0.07	0.30	4.34	−2.45	9.77
	Unclassified	82	−242.22	−376.09	−111.30	−14.18	−22.02	−6.52
	Inland wetlands	51	−8.12	−12.66	−3.32	−15.39	−24.00	−6.30
	Marine	26	−0.31	−1.36	0.47	−3.55	−15.87	5.43
	Mediterranean habitats	33	8.26	−0.86	19.80	20.47	−2.14	49.09
	Montane grassland	5	0.47	0.35	0.58	65.88	48.88	81.27
	Tundra, mires, and moorland	34	−1.97	−2.67	−1.29	−28.56	−38.73	−18.76
	Unknown	2	0.001	0.0002	0.002	29.83	4.71	56.46

**TABLE A3:** Total number (millions of individuals) and estimated change in number (millions, %) of breeding birds in the EU for species with time series derived from national population estimates and national long‐term trends (see Section 2.1.2, *N* = 356), estimated using the imputed model (see Section 2.2.2) and showing the deterministic estimate and the lower and upper bounds of the 95% confidence interval in each case, disaggregated by abundance category

Year	Rare (*N* = 88; 5–16 k)			Scarce (*N* = 89; 16 k–127 k)			Common (*N* = 89; 128 k–1.5 m)			Abundant (*N* = 90; 1.5–222 m)		
	Est	LCL	UCL	Est	LCL	UCL	Est	LCL	UCL	Est	LCL	UCL
1980	0.56	0.52	0.75	5.41	5.13	6.56	59.67	57.99	62.81	1,765.0	1,748.3	1,802.2
2017	0.53	0.52	0.60	5.16	5.07	5.80	48.70	48.10	50.11	1,550.4	1,535.8	1,579.3
Change (m)	−0.02	−0.16	0.004	−0.25	−1.17	0.03	−10.97	−14.03	−8.93	−214.68	−230.75	−202.11
Change (%)	−4.29	−28.02	0.74	−4.55	−21.69	0.62	−18.38	−23.52	−14.96	−12.16	−13.07	−11.45
